# Early definitive internal fixation for infected nonunion of the lower limb

**DOI:** 10.1186/s13018-021-02785-9

**Published:** 2021-10-20

**Authors:** Yong-Cheol Yoon, Chang-Wug Oh, Jae-Woo Cho, Jong-Keon Oh

**Affiliations:** 1 Orthopedic Trauma Division, Trauma Center, Gachon University College of Medicine, Namdong-gu, Incheon, Republic of Korea; 2 Department of Orthopaedic Surgery, School of Medicine, Kyungpook National University, Kyungpook National University Hospital, Jung-gu, Daegu, Republic of Korea; 3 Department of Orthopedic Surgery, Korea University Guro Hospital, College of Medicine, Korea University, 148, Gurodong-ro, Guro-gu, 08308 Seoul, Republic of Korea

**Keywords:** Long bone, Infected nonunion, Staged reconstruction, Early definitive surgery

## Abstract

**Background:**

The management of an infected nonunion of long bones is difficult and challenging. A staged procedure comprising radical debridement followed by definitive internal fixation was favored. However, no standard treatment has been established to determine the appropriate waiting period between initial debridement and definitive internal fixation. We propose a management method that incorporates early definitive internal fixation in infected nonunion of the lower limb.

**Methods:**

Thirty-four patients (28 men and 6 women; mean age 46.09 years; range 25–74 years) with infected nonunion of the tibia or femur were included. Initial infected bone resection and radical debridement were performed in each patient in accordance with the preoperative plans. Definitive surgery was performed 2–3 weeks after the resection (4 weeks after flap surgery was required), and a third surgery was performed to fill the bone defect through bone grafting or transport (three-stage surgery). In cases of unplanned additional surgery, the reason for the requirement was analyzed, and radiological and functional results were investigated in accordance with the Association for the Study and Application of the Method of Ilizarov criteria.

**Results:**

Bone union was achieved in all patients, and treatment was conducted as planned preoperatively in 28 patients (28/34, 82.35%). The mean interval between primary debridement and secondary definitive fixation was 2.76 weeks (range 2–4 weeks). Six unplanned additional surgeries were performed, and the infection relapsed in two cases. The radiological and functional outcomes were good or better in 32 and 31 patients, respectively.

**Conclusions:**

Early definitive surgery can be performed to treat infected nonunion by thorough planning and implementation of radical resection, active response to infection, restoration of defective bones, and soft tissue healing through a systemic approach.

## Background

Infected nonunion of the lower limb is a challenge for even the most experienced surgeons and poses unique problems in controlling florid infections, providing mechanical stability, and early rehabilitation [1]. Less radical approaches can threaten the limb and life of the patient, but invasive approaches can delay bone union [2, 3]. Although few studies have recommended single-stage treatment of infected nonunions, multistage management has gained popularity [4, 5].

Radical debridement, local antibiotic spacer insertion, soft tissue coverage, and temporary stabilization with external fixation are accepted as primary management strategies [6–8]. These are followed by a secondary procedure involving debridement with definitive internal fixation, bone grafting, or distraction osteogenesis.

However, no standard treatment has established the period between initial debridement and final definitive internal fixation, but the mean period ranged from 6 to 8 weeks in previous studies [9–11]_._

The most common problems associated with long external fixation duration are patient distress, pin track infections, soft tissue problems, and delayed healing [12]. Therefore, this study aimed to design an effective staged treatment with early definitive fixation without significant complications for infected nonunion of the lower limb.

## Methods

This study included 34 patients (28 men and 6 women) diagnosed with infected nonunion of the lower limb between March 2013 and December 2016. Each patient underwent a staged reconstructive surgery and was followed up for ≥ 1 year (mean 21.97 months; range 12–53 months). This study was approved by the Institutional Review Board of Korea University Guro Hospital (IRB No. KUGH 13051), and informed consent was obtained from all the patients. All procedures performed in this study involving human participants were in accordance with the ethical standards of the institutional and/or national research committee and with the 1964 Helsinki Declaration and its later amendments or comparable ethical standards.

The mean age of the included patients was 46.09 years (range 25–74 years). The initial causes of trauma were pedestrian accidents (15 cases), car accidents (7 cases), motorcycle accidents (9 cases), trauma due to falls (2 cases), and other causes (sports exercise, one case). The tibia and femur were fractured in 70.59% (n = 24) and 29.41% of the patients (n = 10), respectively. Of the 34 patients, 18 (52.94%) had closed fractures and the other 16 had open fractures. Of the 16 open fractures, 4 were type IIIA, 8 were type IIIB, and 4 were type II, according to the Gustilo and Anderson classification [13]. According to the Cierny–Mader classification, 14 and 20 patients were type-A and type-B hosts, respectively [14]. Seven patients had localized osteomyelitis (type III), and 27 had diffused osteomyelitis (type IV). No type-I or type-II bone involvement was observed (Table 1). Each patient had undergone 2.15 surgeries on average (range 1–5 surgeries) before visiting our clinic. We received cases from all areas of this zone of South Korea as a reference center for septic cases. A plate implant was used in 15 patients, and an Ilizarov external fixator was used in 11. Four patients received intramedullary nail implantation, and four received a monoaxial external fixator.

**Table 1:** Patient demographics

Patient #	Sex	Age	Type of host	Anatomic type of OM	Injury mechanism	Lesion	Site	Fracture classification	# of previous operations	Microorganism
1	M	56	B	Diffused	Pedestrian TA	Tibia diaphysis	Left	Closed	2	MRSE
2	M	36	A	Diffused	Pedestrian TA	Tibia metaphysis (distal one-third)	Right	IIIA	2	Culture negative
3	M	43	B	Diffused	Sports exercise	Tibia metaphysis (distal one-third)	Left	Closed	5	*Enterobacter cloacae*
4	M	45	B	Diffused	Pedestrian TA	Femur diaphysis	Right	Closed	2	Culture negative
5	M	49	B	Diffused	Motorcycle TA	Tibia diaphysis	Right	IIIB	3	*Pseudomonas*
6	M	61	B	Diffused	In car TA	Femur diaphysis	Left	Closed	2	MRSE
7	M	61	B	Diffused	In car TA	Both tibia Lt: Diaphysis Rt: Tibia Metaphysis	Both	IIIB	2	*Acinetobacter baumannii*
8	M	61	B	Diffused	Motorcycle TA	Tibia metaphysis (Distal one-third)	Right	IIIA	1	MRSA
9	M	25	A	Localized	Motorcycle TA	Tibia diaphysis	Right	IIIA	2	*Pseudomonas*
10	M	58	B	Diffused	Motorcycle TA	Tibia metaphysis (Distal one-third)	Left	IIIB	1	Culture negative
11	F	40	B	Diffused	In car TA	Both tibia Lt: Diaphysis Rt: Metaphysis (Distal one-third)	Both	IIIB	1	*Acinetobacter baumannii*
12	M	50	B	Diffused	Motorcycle TA	Tibia metaphysis (Distal one-third)	Right	Closed	3	MRSA
13	M	68	B	Localized	In car TA	Tibia metaphysis (Proximal one-third)	Right	II	1	*#1. Acinetobacter baumannii* *#2. Enterobacter cloacae* *#3. Stenotrophomonas maltophilia*
14	F	28	A	Localized	In car TA	Femur diaphysis	Right	Closed	1	MRSA
15	F	30	A	Diffused	Fall	Femur diaphysis	Right	Closed	3	*Enterococcus faecium*
16	M	64	B	Diffused	Pedestrian TA	Tibia metaphysis (Distal one-third)	Right	Closed	2	MSSA
17	M	74	B	Diffused	Motorcycle TA	Femur diaphysis	Left	Closed	2	Culture negative
18	M	35	A	Localized	Motorcycle TA	Femur diaphysis	Right	Closed	4	*Enterococcus gallinarum*
19	F	25	A	Diffused	Fall	Femur diaphysis	Right	Closed	3	Culture negative
20	M	33	A	Diffused	Pedestrian TA	Tibia metaphysis (Distal one-third)	Right	IIIB	2	MRSA
21	M	34	A	Diffused	Pedestrian TA	Tibia diaphysis	Right	Closed	1	*Enterococcus faecalis*
22	M	30	A	Diffused	Pedestrian TA	Tibia diaphysis	Right	IIIB	5	*Serratia marcescens*
23	M	51	B	Localized	Pedestrian TA	Femur metaphysis (Distal one-third)	Left	Closed	2	Culture negative
24	M	67	B	Diffused	Pedestrian TA	Tibia metaphysis (Distal one-third)	Left	IIIB	2	Culture negative
25	M	51	B	Diffused	Pedestrian TA	Tibia metaphysis (Distal one-third)	Left	Closed	1	Culture negative
26	M	36	A	Localized	Pedestrian TA	Tibia metaphysis (Distal one-third)	Right	IIIA	5	*Pseudomonas*
27	F	59	B	Localized	Pedestrian TA	Tibia diaphysis	Left	Closed	2	MSSA, *Escherichia coli*
28	M	59	B	Diffused	Motorcycle TA	Tibia metaphysis (Proximal one-third)	Right	Closed	3	MRSA
29	M	28	A	Diffused	In car TA	Femur metaphysis (Distal one-third)	Right	II	1	*Pseudomonas*
30	M	25	A	Diffused	Pedestrian TA	Tibia diaphysis	Left	IIIB	1	Culture negative
31	F	51	B	Diffused	Pedestrian TA	Tibia diaphysis	Left	II	2	MRSE
32	M	44	A	Diffused	In car TA	Femur diaphysis	Right	Closed	1	MSSA
33	M	45	A	Diffused	Motorcycle TA	Tibia diaphysis	Left	Closed	2	Culture negative
34	M	45	B	Diffused	Pedestrian TA	Tibia diaphysis	Right	II	1	MSSA

#: number, M: male, F: female, TA: traffic accident. OM: osteomyelitis, MRSA: methicillin-resistant *Staphylococcus aureus*, MSSA: methicillin-susceptible *Staphylococcus aureus*, MRSE: methicillin-resistant *Staphylococcus epidermidis*

Infected nonunion was defined as failure to attain bony union within 6–8 months after the initial injury, with infection localized to the nonunion site [1, 5]. Patients were diagnosed with an infection when an open wound or sinus tract was present before surgery, pus was found intraoperatively, and the causative organism was identified. The patients’ medical histories were carefully examined, and surgery or fracture site was checked for rash, edema, and/or fever. In addition, hematological examination for parameters, including white cell count (WBC), erythrocyte sedimentation rate (ESR), and C-reactive protein (CRP), was performed when nonunion occurred despite multiple surgical treatments, after treatment of the initial open fracture, or when the patient underwent long-term fixation using an external fixator during the initial treatment [1, 5, 6]. Radiography, computed tomography, and magnetic resonance imaging of the nonunion site were conducted to evaluate the state of the cortical bone, the presence and range of sequestrum, any changes in the osseous tissue around the internal fixator, and the extent of soft tissue infection. A nonunion site suspected of infection was opened and checked [4, 15]_._

### Treatment strategies

**Fig. 1:**
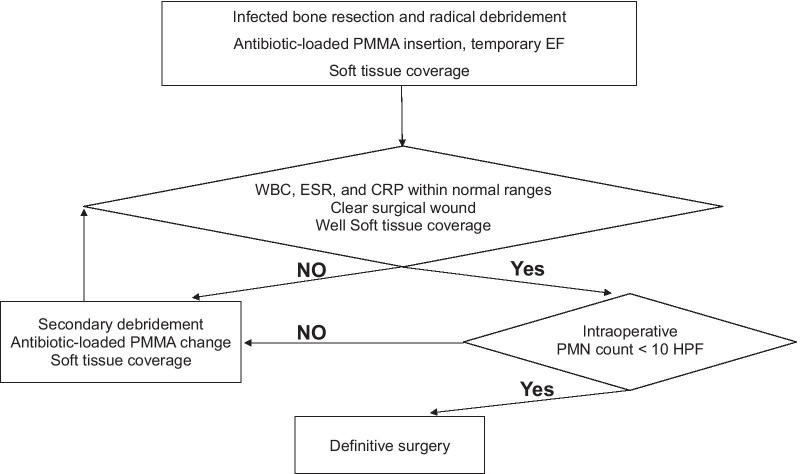
Treatment algorithm for infected nonunions

All patients with infected nonunion underwent surgery in accordance with a three-stage standard treatment (Fig. 1). The first stage involved the removal of all internal fixations (if any) and a systematic radical debridement of soft tissue, followed by resection of the sequestered bone, in accordance with the preoperative plan. The debridement had to be meticulous, as the quality of debridement seemed to be the most important factor in the success of the procedure. Debridement was performed until all pale-looking tissues were excised. A safe margin for bone resection was determined when active bleeding in the bone remaining after resection was confirmed by the “paprika sign.” Routine irrigation was performed using a pulsatile lavage system as described in a previous study, and the drapes were changed [16]. We used 4 g of vancomycin with 40 mg of polymethyl methacrylate (DePuy CMW 3 gentamicin bone cement, DePuy Synthes, Raynham, MA, USA) to fill the gap between the resected ends of the bone. A monoaxial external fixator was used for temporary bone fixation. We sutured the soft tissue with minimal tension; however, for cases requiring soft tissue reconstruction because of the extensive soft tissue defect, flap surgery was performed under consultation with a plastic surgeon from the preoperative planning step. A rotational flap was preferred over a free flap owing to the advantages of its high survival rate and short duration of surgery, except in cases with poor soft tissue condition and extensive defects that could not be covered by the rotational flap [17]. In cases where it was difficult to perform a flap surgery immediately, vacuum-assisted closure (VAC, KCI, San Antonio TX, USA) was performed, followed by immediate soft tissue reconstruction [18].

During the period between the primary and secondary procedures, we checked the WBC, ESR, and CRP levels once every 3 days [19]. Definitive surgery was planned when the WBC, ESR, and CRP levels were within their normal ranges and soft tissue coverage was adequate with good flap uptake. In consultation with an infectious disease physician, appropriate antibiotics were administered according to the microbe identified from the initial surgery, and the timing of the definitive surgery (not later than 2–3 weeks after resection) was determined by considering the patient’s physical condition, fever, and nutritional status. If the patient had a sustained fever for 3 weeks, non-decreasing hematological markers, or worsened wound condition, repetitive debridement was performed. In cases requiring extensive soft tissue coverage with free or localized flaps, to avoid flap margin necrosis, definitive fixation was delayed by 3–4 weeks following the recommendation of the plastic surgeon.

The second stage comprised thorough debridement, intraoperative polymorphonuclear (PMN) leukocyte count evaluation, and internal fixation with vancomycin-loaded cement spacer insertion. The internal fixator was fixed with an external fixator in situ for bone transport in 14 cases; in the remaining 20 cases, the external fixator was removed, and only the internal fixator was retained for an induced membrane technique [5]. Internal fixation with an intramedullary nail was performed; however, we resorted to plating in cases where one fragment of bone was extremely short for nailing.

Infection was presumed to be eradicated when the intraoperative PMN count in a frozen biopsied section of representative tissue was < 10/high-power field (HPF) [20]. We confirmed the absence of infection by calculating the intraoperative PMN count in frozen biopsied sections from five representative zones in each patient (proximal intramedullary, proximal extramedullary, central, distal intramedullary, and distal extramedullary) and proceeded with definitive internal fixation. If the PMN count was > 10/HPF in any zone, the patient underwent secondary debridement, soft tissue coverage, and changes in antibiotic beads/spacers, and definitive fixation was deferred.

After the procedure, active range-of-motion exercises assisted by mechanical continuous passive motion devices were encouraged. The patients were counseled to be active and ambulatory. Intravenous antibiotics were administered for an additional 4–6 weeks, in accordance with the culture reports, and the WBC, ESR, and CRP levels were monitored weekly. The patients were discharged when the wound around the surgery site was in good condition with normal findings on examinations of the inflammatory markers, and were observed in follow-up sessions every month.

The final stage of the treatment included bone graft surgery, as excision of the involved bone segment during radical debridement resulted in a bone defect. We used one of the two methods to fill the gap during the period between the second- and third-stage surgeries, depending on the location and size of the defect and the condition of the soft tissue. If the gap was < 4–6 cm in both the tibia and femur, the bone defect was judged to be in the metaphysis, or if the long-term installation of the external fixator was challenging because of the poor condition of the soft tissue, we used the induced membrane technique with massive bone grafting to fill the defect [21, 22]. During the second-stage surgery, antibiotic-impregnated cement was placed around the internal fixation site to bridge the defect between the fragments. We waited 8–10 weeks for the membrane to completely form and then performed the third stage, which involved morcellized bone grafting from the anterior or posterior iliac crest into the membrane after removing the cement. When the amount of bone graft was insufficient, circumferential bone grafting was performed with the absorbable gelatin sponge as a core [23].

We used distraction osteogenesis for defects > 4–6 cm in size. We chose bone transport over a nail or plate to fill the gap in the bone when the defect was in the diaphysis or the condition of the soft tissue in the defect site was good [24]. During the period between the second- and third-stage surgeries, the patients were educated on the method of self-adjusting the length of the external fixator bars to move the bone by 1 mm/day. All the patients who underwent bone transport received bone grafting and augmentation plate fixation as the third-stage surgery.

Bone union during the follow-up period (> 1 year) was monitored depending on the final surgery of the patients. Bone union and functional outcomes were determined based on the Association for the Study and Application of the Method of Ilizarov (ASAMI) criteria [24, 25] (Table 2). The number of patients who had a planned definitive internal fixation, the number of patients who required additional surgeries, and the reason for those additional surgeries were recorded.

**Table 2:** Association for the Study and Application of the Method of Ilizarov (ASAMI) classification

	Bony results	Functional results
Excellent	Union, no infection, deformity < 7°, and limb length discrepancy < 2.5 cm	Active, no limp, minimum stiffness (loss < 15° knee extension/15° ankle dorsiflexion), reflex sympathetic dystrophy (RSD), and insignificant pain
Good	Union + any two of the following: absence of infection, deformity < 7°, and limb length discrepancy of 2.5 cm	Active with one or two of the following: limp, stiffness, RSD, and significant pain
Fair	Union + one of the following: absence of infection, deformity < 7°, and limb length discrepancy of 2.5 cm	Active with three or all of the following: limp, stiffness, RSD, and significant pain
Poor	Nonunion/re-fracture/union + infection + deformity of 7° + limb length discrepancy of 2.5 cm	Inactive (unemployment or inability to return to daily activities due to injury)

## Results

Bone union was achieved in all 34 patients, as observed during the follow-up (> 1 year). In 22 cases, early definitive surgery was performed within 2–3 weeks of resection, as shown in the treatment proposed in this study. Among the total of 6 cases of flap surgery performed because of the soft tissue defect, a rotational flap was used in 5 cases and a free flap was used in 1 case (Table 3). Flap surgeries were performed within 4 weeks after the initial surgery. Therefore, the treatment was performed as planned preoperatively in 28 patients (28/34, 82.35%). Considering the soft tissue coverage and any additional surgeries, the mean interval from the initial surgery to the definitive surgery was 2.76 weeks (range 2–4 weeks). Six unplanned additional surgeries were performed, and secondary debridement was required in four cases (two because of increased CRP level before the definitive surgery and two because the PMN count was ≥ 10/HPF). We repeated the debridement and waited for another 2 weeks for reimplantation of the internal fixation. Even in these cases, definitive fixation was completed within 4 weeks. The infection recurred in two patients (patients 3 and 15) and 2 and 6 months after the definitive surgery, respectively. In these cases, all the implants were removed, a secondary debridement was performed, and another definitive surgery was performed using an Ilizarov external fixator (patient 3) or intramedullary nailing (patient 15) after 2–3 weeks.

**Table 3:** Treatment result of infected nonunion

Patient #	Antibiotics	Previous implant	DS implant	Interval to DS (weeks)	Additional operation Why? How?	Bone defect size (cm)	Bone graft or transport	Radiological result	Functional result	Union time (weeks)	F/U (months)
1	Vancomycin	IM nail	IM nail	2	None	2.5	G	Excellent	Excellent	16	12
2	Cephalosporin	Plate	Plate	2	None	5.2	T	Excellent	Excellent	24	53
3	Tazocin	Plate	Plate	3	Recurrence of infection Implant removal Secondary debridement Changing of cement beads Ilizarov application (3 wks later)	4.8	T	Fair	Fair	52	27
4	Cefazolin	IM nail	IM nail	4	Intra op PMN > 20 HPF Secondary debridement Changing of cement beads IM nail (2 wks later)	7.2	G	Good	Good	20	12
5	Ceftazidime	Monofixator	Plate	4	Soft tissue defect Gastrocnemius Rotation flap Plate (2 wks later)	2.5	G	Good	Fair	20	27
6	Teicoplanin	IM nail	IM nail	3	None	5.8	G	Excellent	Excellent	18	33
7	Imipenem	Monofixator	I + P	4	Soft tissue defect Gastrocnemius Rotation flap IM nail (2 wks later)	5.2	T	Excellent	Good	24	24
8	Vancomycin	Monofixator	I + P	2	None	5.9	T	Good	Good	16	12
9	Cefepime + ciprofloxacin	Ilizarov	IM nail	2	None	3.1	G	Excellent	Excellent	16	12
10	Cephalosporin	Ilizarov	IM nail	2	None	4.6	G	Excellent	Excellent	22	14
11	Meropenem	Rt: Ilizarov Lt: Monofixator	I + P	2	None	4.5	T	Excellent	Good	16	28
12	Teicoplanin	Ilizarov	I + P	2	None	3.2	T	Good	Good	35	15
13	Cravit	Plate	Plate	2	None	5.6	T	Excellent	Excellent	20	30
14	Vancomycin	Plate	IM nail	3	None	2.3	G	Good	Good	20	12
15	Cefazedone	Plate	IM nail	3	Recurrence of infection Implant removal Secondary debridement Changing of cement beads IM nail (2 wks later)	3.2	G	Fair	Fair	20	17
16	Vancomycin	Plate	IM nail	2	None	4.1	G	Good	Good	24	37
17	Cravit + Rifampin	IM nail	IM nail	2	None	1.8	G	Excellent	Excellent	20	12
18	Unasyn	Plate	IM nail	4	Intra op PMN > 20 HPF Secondary debridement Changing of cement beads IM nail (2 wks later)	4.1	G	Fair	Fair	20	24
19	Cefaclor	Plate	IM nail	2	None	2.2	G	Excellent	Good	24	24
20	Gentamycin	Plate	I + P	4	Soft tissue defect Gastrocnemius Rotation flap I + P (2 wks later)	10.2	T	Excellent	Excellent	20	30
21	Cycin + Unasyn	Monofixator	IM nail	2	None	6.1	G	Good	Good	18	16
22	Ciprofloxacin	Plate	I + P	4	Soft tissue defect Rotation flap surgery IM nail (2 wks later)	10.3	T	Excellent	Excellent	20	12
23	Cefotaxime	Plate	Plate	2	None	2.1	G	Excellent	Excellent	12	12
24	Cefotaxime	Ilizarov	IM nail	4	Soft tissue defect Distally based hemisoleus flap I + P (2 wks later)	2.9	G	Excellent	Excellent	12	12
25	Cefotaxime	Ilizarov	I + P	2	None	5.2	T	Excellent	Excellent	20	12
26	Ciprofloxacin	Ilizarov	I + P	2	None	7.8	T	Excellent	Excellent	12	12
27	Ertapenem	Ilizarov	IM nail	3	None	1.7	G	Excellent	Excellent	12	48
28	Teicoplanin	Ilizarov	Plate	2	None	3.5	T	Excellent	Excellent	20	24
29	Ciprofloxacin	Ilizarov	Plate	2	None	5.4	T	Good	Good	20	24
30	Cefotaxime	Ilizarov	I + P	4	Soft tissue defect ALT flap surgery IM nail (2 wks later)	4.8	T	Good	Good	24	24
31	Teicoplanin	Plate	Plate	4	CRP = 40 mg/L (0 ~ 5 mg/L) Secondary debridement Changing of cement beads Plate (2 wks later)	1.7	G	Excellent	Excellent	24	12
32	Cefotaxime	Plate	IM nail	3	None	1.6	G	Excellent	Excellent	24	12
33	Cefotaxime	Plate	Plate	4	CRP = 50 mg/L Secondary debridement Changing of cement beads Plate (2 wks later)	1.5	G	Good	Excellent	12	24
34	Cefaclor	Plate	Plate	2	None	1.4	G	Excellent	Excellent	20	48

IM: intramedullary, DS: definitive surgery, PMN: polymorphonuclear leukocyte, I + P: intramedullary nailing with augmented plate

HPF: high-power field, ALT: anterolateral thigh, CRP: C-reactive protein, G: bone graft, T: bone transport

**Fig. 2:**
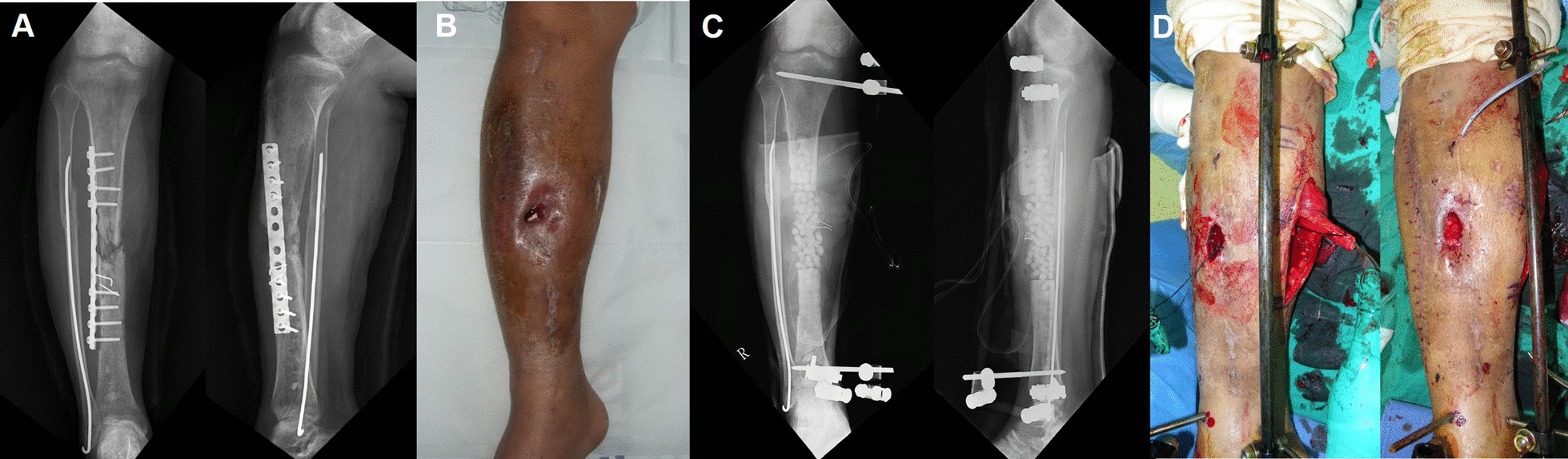
**a, b** A 33-year-old man (case 20) diagnosed with a right tibiofibular shaft open fracture (Open type IIIB) underwent open reduction surgery with internal fixation using plating at another institution. Continuous discharge and formation of a sinus tract were observed at the surgery site postoperatively. **c** To treat this, infected bone resection and radical debridement were carried out, antibiotic-coated beads were inserted, and a temporary external fixator was installed at our hospital. **d** Gastrocnemius rotation flap surgery was carried out for the soft tissue defect

**Fig. 3:**
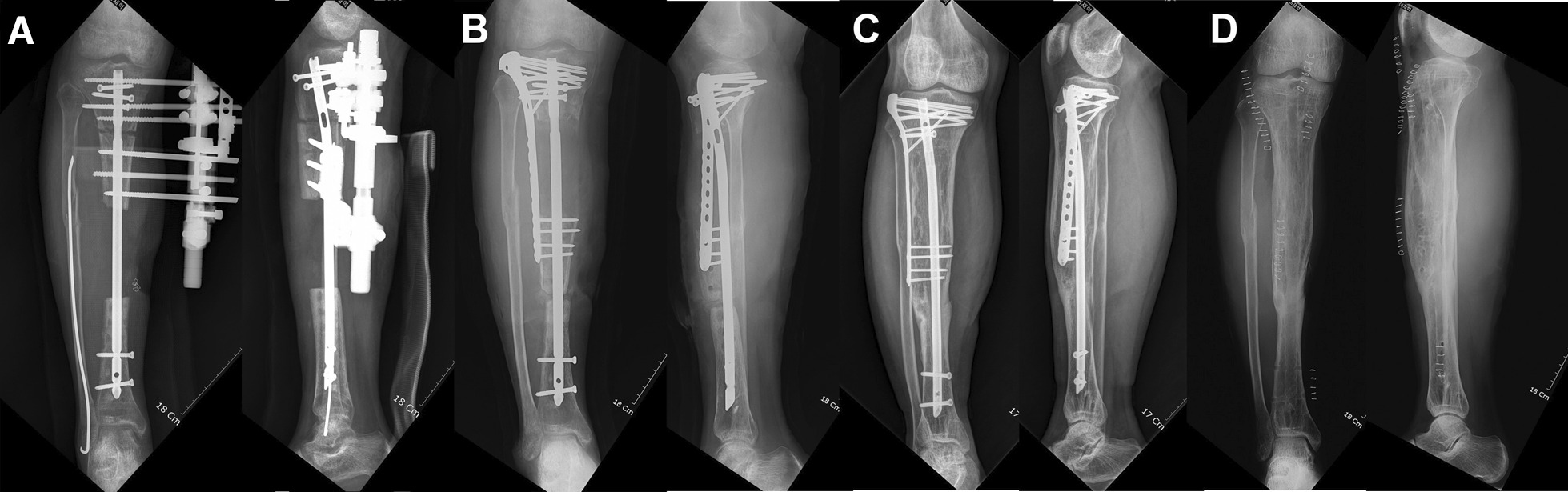
**a** The patient underwent treatment as per the preoperative plan, and bone transport was done over the nailing that secured the fractured bones which was performed as definitive surgery. **b** Three months post-surgery, docking site bone grafting and augmentation plating were performed. **c, d** Bone union was achieved, and implant removal was performed 1 year post-surgery without complications

The mean bone defect size was 4.24 cm after the initial radical debridement (range 1.4–10.3 cm). The bony defect site was filled by bone transport in 14 patients and bone grafting in 20 patients. In the definitive surgery, fixation was performed with intramedullary nailing in 23 patients and with plates in 11 patients. In nine patients, docking site bone grafting with augmented plating was performed after bone transport over nailing (Figs. 2, 3).

The mean duration of fracture healing was 20.5 weeks (range 12–52 weeks). The radiological and functional results obtained from the completed follow-up were evaluated using the ASAMI classification. A radiological result of “Excellent” was achieved in 21 patients. A “Good” result was achieved in 10 patients, and a “Fair” result was achieved in 3 patients (Table 3). A functional result of “Excellent” was achieved in 19 patients: “Good” in 11 and “Fair” in 4.

## Discussion

In this study, a high treatment success rate was achieved in infected nonunion, which is known for difficult radical resection and long treatment duration, by confirming radical debridement on the basis of the PMN count and three-stage surgery through early definitive fixation.

Many studies have suggested that radical debridement is the most important factor in the success of nonunion surgery [8, 26, 27]. However, no consensus has been reached regarding the method or time at which radical debridement should be performed. The “paprika sign,” characterized by punctate cortical or cancellous bleeding, is the most common indicator that further debridement is necessary [28]_._ However, punctating all infected bones is not possible, and checking for bleeding may be difficult because of the condition or anatomical position of the cortical bone. Therefore, we introduced the PMN count, which, in conjunction with the paprika sign, confirmed the presence of infection during arthroplasty surgery [20, 29]. In our study, after removing the infected bone, the five most likely sites of infection in the margin were selected, and the PMN count was determined to confirm whether the infection was completely eliminated. This resulted in a significantly reduced infection recurrence rate of 5.88% (2/34).

Struijs et al. conducted a systematic review of 16 articles about one-stage revision surgery and 18 articles about two-stage revision surgery for infected nonunion and reported that the highest union rate and the lowest persistent infection rate were achieved with a method that included debridement, antibiotic beads, and planned secondary fixation [5]. Motsitsi reported that staged surgery was the most effective treatment based on research studies conducted between 1996 and 2006, and “infection elimination” is the first stage in any treatment [8]_._ In addition, we showed that a three-stage surgical strategy is safe and effective for preventing infection recurrence. Some authors have attempted to treat infection and nonunion simultaneously through a single-stage surgery; however, treating nonunions after establishing the optimal conditions for bone union by treating the infection first is considered to be inadequate because of the high recurrence rates [1, 30]_._ Two-stage surgery, in which definitive internal fixation and bone grafting are performed simultaneously, has also been performed [31, 32]_._ However, even in this two-stage surgery, there is no objective evidence that the infection has been removed, the amount of autologous bone that can be filled with bone is limited, and complications related to the bone graft cannot be neglected.

Although numerous studies have reported successful treatment of infected nonunions with staged surgery and an interval between the initial and second surgeries of 6–8 weeks [9–11, 33]_,_ we could not find a clear scientific reasoning for this interval. The long interval could be due to the tendency to expect a higher therapeutic effect by increasing the duration of systemic intravenous antibiotic administration. However, an interval of > 6 weeks inevitably results in more problems, including the risk of pin-site infection or pin loosening due to external fixators [12]. Consequently, a vicious cycle with resumption of intravenous administration of antibiotics was repeated to resolve these problems.

Earlier definitive internal fixation leads to shorter treatment periods and shorter hospital stays, which inevitably results in a lower economic burden for the patient. In addition, it enables earlier weight bearing and shorter rehabilitation effectively, resulting in faster muscle strength recovery and improved quality of life. Therefore, treatment of infected nonunion by radically removing the lesions and administering appropriate antibiotics against any infection remaining after surgical treatment is critical. However, a definitive surgery does not need to be performed within 2–3 weeks after the initial surgery if patients exhibit fever, swelling, and/or redness at the surgery site, if hematological abnormalities (increase in WBC, ESR, and CRP) are present, or if the intraoperative PMN count is ≥ 10. However, this does not mean that the surgeon should wait to administer intravenous antibiotics for a long time. In these cases, the areas suspected of infection should be actively removed through secondary debridement. Either definitive surgery or secondary debridement should be performed to treat the infection origin actively, except in cases requiring flap surgery.

The following are the two general methods of filling bone defects: bone grafting using the induced membrane technique and bone transport using distraction osteogenesis. We chose one of the two methods for each patient by considering the size and location of the bone defect and the condition of the surrounding soft tissue [1, 22, 30]. Bone grafts limit the amount of bone that can be collected, and donor-site complications may occur. Also, for bone tissue regeneration, extracorporeal shock wave therapy, ultrasound, electromagnetic, bone morphogenic proteins, and platelet-rich plasma have been introduced in addition to autologous bone. Still, an apparent therapeutic effect cannot be guaranteed [34]. Conversely, bone transports have the disadvantages of pin-site infection and joint contraction, which may result from long-term installation of external fixators. Therefore, surgeons should be aware of the advantages and disadvantages of each technique when choosing the optimal method for their patients. Rigal et al. reported good clinical outcomes when using a bone defect standard of 6 cm when choosing either bone graft or transport [35]. Based on our clinical outcome, we believe that using either method is reasonable, depending on the individual needs of the patients.

Our study has several limitations. First, a relatively small number of patients were enrolled because only those with infected nonunion were evaluated, and no control group was included. Second, the relatively short follow-up period might have limited our evaluation of clinical outcomes regarding recurrence rate in patients who were followed up for > 1 year. Nonetheless, we believe that our findings provide sufficient evidence to support the effectiveness of a staged surgical treatment in cases with severe complications from infected nonunion occurring within 1 year postoperatively. This retrospective study investigated the effectiveness of staged surgery based on clinical and radiological results. Future prospective studies should be conducted to validate our results.

## Conclusions

Waiting is not a solution when treating infected nonunions. Early definitive surgery is possible with careful planning and implementation of radical resection of infected bone, active response to infection, reconstruction of the defective bone, and soft tissue healing through a comprehensive and systemic approach.

## Data Availability

The datasets generated and/or analyzed during the current study are not publicly available due to the restricted access to our hospital database but are available from the corresponding author on reasonable request.

## Abbreviations

WBC: White cell count
ESR: Erythrocyte sedimentation rate
CRP: C-reactive protein
VAC: Vacuum-assisted closure
PMN: Polymorphonuclear
HPF: High-power field
ASAMI: Association for the Study and Application of the Method of Ilizarov
